# Supplementary material for: Ultrasensitive mechanical/thermal response of a P(VDF-TrFE) sensor with a tailored network interconnection interface
Source: Nat Commun. 2023 Jul 6;14:4000. doi: 10.1038/s41467-023-39476-4 (PMC10326000; doi:10.1038/s41467-023-39476-4)
Supplement: Supplementary file 1 — Supplementary information [file 41467_2023_39476_MOESM1_ESM.pdf]

Ultrasensitive Mechanical/Thermal Response of a P(VDF-TrFE) Sensor with a Tailored Network Interconnection Interface

Bo Li[1]*, Chuanyang Cai[2], Yang Liu[3], Fang Wang[4], Bin Yang[1], Qikai Li[1], Pengxiang Zhang[1], Biao Deng[1], Pengfei Hou[2]*, Weishu Liu[1]*

[1]Department of Materials Science and Engineering, Southern University of Science and Technology, Shenzhen, Guangdong 518055, China

[2]School of Materials Science and Engineering, Xiangtan University, Hunan Xiangtan 411105, China

[3]State Key Laboratory of Material Processing and Die & Mould Technology, School of Materials Science and Engineering, Huazhong University of Science & Technology, Wuhan 430074, China

[4]Institute of Biomedical & Health Engineering, Shenzhen Institute of Advanced Technology (SIAT), Chinese Academy of Sciences (CAS), Shenzhen, 518055, China.

*E-mail: lib6@sustech.edu.cn; houpf@xtu.edu.cn; liuws@sustech.edu.cn

This PDF file includes:
Discussion
Fig. S1 to S20
Tables S1 to S5
References

Discussion

Analysis of the increase in the piezoelectric coefficient (d_{33}) attributable to NII

1) Phase content: the d_{33} of the P(VDF-TrFE) film depends on its β-phase content. For the P(VDF-TrFE) and P(VDF-TrFE)/PEDOT:PSS composite film, X-ray diffraction shows that the strongest diffraction at 2θ=20.5 °C of β-phase with (110)/(200) planes, as shown in Fig. S2B. The Bragg diffraction intensity of P(VDF-TrFE) is greater than that of the P(VDF-TrFE)/PEDOT:PSS composite film, indicating that the β-phase phase content does not increase.

2) PEDOT:PSS electrode: the d_{33} of the PEDOT:PSS electrode without P(VDF-TrFE) film was measured with ZJ-3AN, and therefore the PEDOT:PSS electrode does not contribute to the improvement of the d_{33}.

3) Thickness: the d_{33} of the P(VDF-TrFE) film depends on its thickness, and the P(VDF-TrFE) film used in this work holds 80 μm. The thickness of the P(VDF-TrFE) film does not contribute to the d_{33}.

The pyroelectric coefficient of composite film obtains for experimental

The pyroelectric coefficient of the composite film can be determined by the following equation:

$$p=\frac{I}{A\cdot dT/dt} \quad (1)$$

where I is the current, A is the area of electrode, T is the temperature, and t is the time. Measured data used to calculate the pyroelectric coefficient in the P(VDF-TrFE)/PEDOT:PSS composite film was listed in Table. S2.

Calculation of piezoelectric and pyroelectric voltage output of the devices

The piezoelectric voltage output of the devices (S_p) can be obtained from the equation of $S_p=\Delta V_p/\Delta P$, where ΔV_p and ΔP are the relative change of piezoelectric voltage output and applied pressure, respectively[1]. The pyroelectric voltage output of the devices $S_t=\Delta V_t/\Delta T$, where ΔV_t and ΔT are the relative change of pyroelectric voltage output and temperature, respectively.

Coefficient of determination

R^2 in the manuscript indicates the degree of fit of the trend line indicator, and its numerical magnitude reflects the degree of fit between the estimated value of the trend line and the corresponding actual data. The higher the degree of fit, the more reliable the trend line is. The R-squared value is calculated as follows. The R-squared value is calculated as follows[2,3]:

$$R^2=\frac{SSR}{SST}=\frac{\sum_{i=1}^{n}(\hat{y}_i-\bar{y})^2}{\sum_{i=1}^{n}(y_i-\bar{y})^2} \quad (2)$$

where $\hat{y}_i$ is the voltage, and $\bar{y}$ is the average voltage.

Theoretical analysis of response time of piezoelectric sensor from domain movement

To study the influence of the NII on the response time of piezoelectric signal, the phase field simulation is applied to simulate the movement of the domain under pressure. In the phase field simulation, the total number of time steps represents the development rate of the domain structure of P(VDF-TrFE) under the external field. The larger value of the time step, the slower the evolution rate will be.

The simulated and actual domain evolution time can be determined by the kinetic coefficient (L) in the TDGL equation, which can be obtained for domain wall dynamics experiment. Since the value of L is not determined, the normalized time and polarization

is used in Fig. S11. The simulation results show that the evolution time of the P(VDF-TrFE) film with NII under external forces (133 steps) is shorter than that of the film without NII (276 steps), which is in line with our experimental results. Therefore, the response time of the P(VDF-TrFE) with PEDOT:PSS electrode is lower than that of the metal electrode, which can be explained to some extent from the perspective of domain movement.

Actually, a piezoelectric sensor consists of an electrode, piezoelectric materials and an interface between them. This means that these factors can affect the response time of the piezoelectric sensor. This theoretical analysis of the response time of the piezoelectric sensor only considers the movement of the domain under mechanical load[4], the influence of other factors, such as the electrode materials and microstructures, should be further investigated.

The PDMS has no piezoelectric properties and cannot induce voltage under the pressure. When a stress applied to our devices encapsulated with PDMS, the deformation can be enlarged and increase the piezoelectric voltage. In this work, the thickness of the PDMS was 200 μm, and it can be considered that it cannot enhance the piezoelectric voltage. The PEDOT:PSS was an electrode that also has no piezoelectric effect and increases the voltage.

How to separate the contribution of piezoelectric and pyroelectric effects in the pressure and temperature stimuli

1) Voltage was measured using a bottle of water at room temperature (25 °C). The temperature of the water in the bottle was measured using a thermocouple to ensure that it was the same as the ambient temperature and that no pyroelectric voltage was generated. Therefore, it can be assumed that this voltage is generated only by the piezoelectric effect (U_{pi}).

2) The water in the bottle was heated to different temperatures (usually 30, 35 and 40 °C), and then the voltage was measured using the 6514. In this case, both the piezoelectric and pyroelectric effects contribute to the measured voltage (U_{to}). The contribution of the pyroelectric voltage (U_{py}) to the total voltage can be determined by

U_{py}=U_{to}-U_{pi}. Then a proportionality factor (P_{pipy}) between the pyroelectric and pyroelectric effect is defined as $P_{pipy} = U_{pi} : U_{py}$.

3) If the devices were used to measure temperature and pressure simultaneously, the contribution of the pyroelectric effect and the piezoelectric effect to the voltage can be determined by P_{pipy}.

4) The piezoelectric and piezoelectric voltage curves show that the response time of these two effects is different. This feature has been found to be helpful to show the pyroelectric effect and the piezoelectric effect.

It should be noted that the third coefficient, related to the piezoelectric effect, results from the temperature gradient along the polar axis of the ferroelectrics[4]. The piezoelectric stress can contribute to the pyroelectric voltage when the sample is larger. In this work, pyroelectric is mainly considered as a temperature monitoring application. We have assumed that the influence of piezoelectric effect on pyroelectric voltage has little effect on temperature monitoring.

Application of sensor for real-time monitoring

Loading–unloading experiments were applied to verify that the composite film can detect pressure and temperature stimuli as a sensor. Pressing and release of a 100 g glass bottle on the sensor at 0.2-0.75 Hz yielded the findings in Fig. S12a. Pressure indications alone are obtained, with no temperature change. Fig. S12b shows the thermal response signals without a pressure signal when the device was periodically heated (0.1-0.5 Hz) by a noncontact hot air source (temperature difference of 2 °C). Fig. S12c shows that the device simultaneously generates piezoelectric and pyroelectric voltages when in contact with a finger due to the mechanical pressure caused by pressing and the fingertip temperature. Fig. S12d shows the "press-hold-release" and "press-pressing-release" waveforms of the device, the differences were present in supplementary information. This result shows that the composite film can detect several body movements.

Fig. S13c shows the response of the sensor attached to the subject's fingertip when objects with different weights were picked up. Both the pressure voltage (obtained from 6517B) and temperature voltage (obtained from 6514) generated by the device vary according to

the object being carried. This is because a greater force is required to hold a heavy object steady, and the pressure applied to the device is thus increased. The temperature signals are similarly modified as the temperature radiated by the fingertip varies according to the magnitude of the force being exerted. When the external force applied to the object increases, the contact area between n the sensor and the object also increases, increasing the heat exchange per unit time and decreasing the interface thermal resistance due to the elasticity of the rubber glove and skin. As a result, $\mathrm{d}T/\mathrm{d}t$ increases and leads to an increase in pyroelectric voltage and make the voltage increase with increase of pressure, as shown in Fig. S13c.

Figures

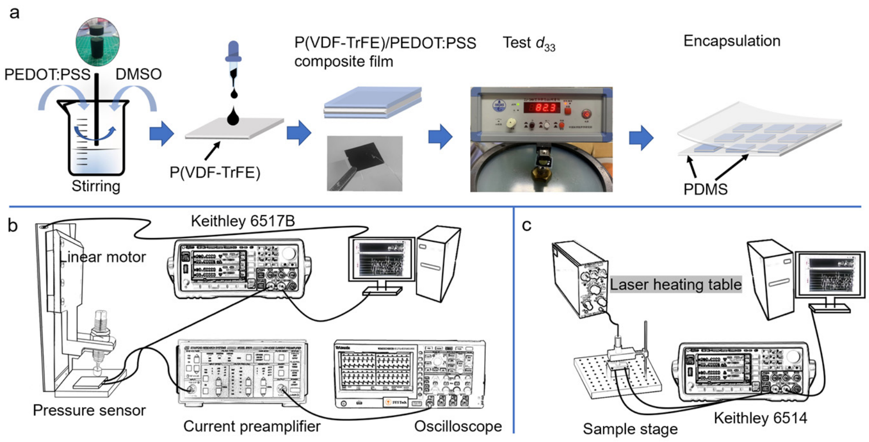

Fig. S1 Schematic diagram of the preparation and testing procedure. (a) Preparation of the P(VDF-TrFE)/PEDOT:PSS composition film and fabrication of the sensor device. The setup of (b) pyroelectric and (c) piezoelectric output test system.

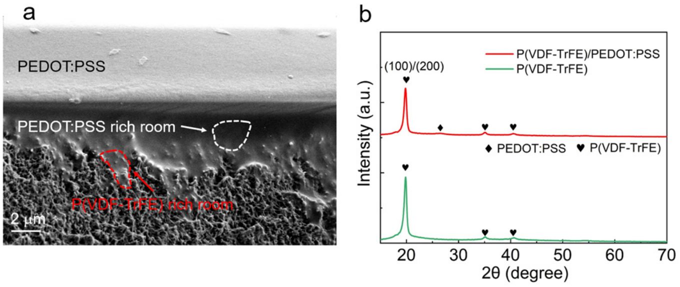

Fig. S2 The microstructure and X-ray image of the composite film. (a) SEM image of the cross-section of the PEDOT:PSS/P(VDF-TrFE) composite film. (b) X-ray diffraction of P(VDF-TrFE) with and without PEDOT:PSS

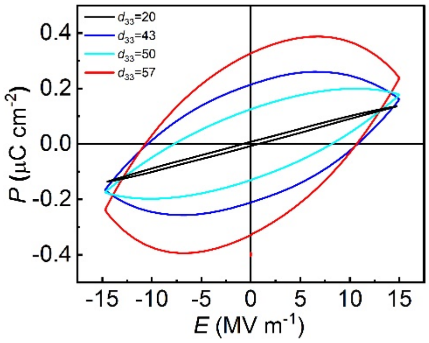

Fig. S3 Polarization-electric field hysteresis loops of the P(VDF-TrFE) film with Au electrode (d_{33}=20) and PEDOT:PSS electrode(d_{33}=43, 50, 57).

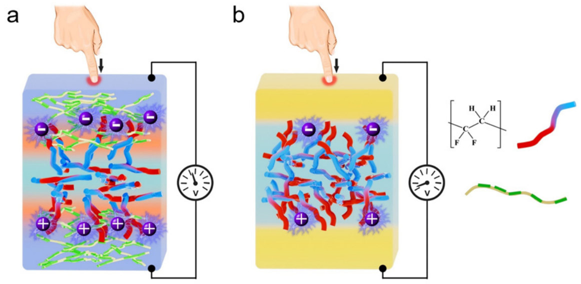

Fig. S4 Schematic diagram of the charge collection in the structure of (a) P(VDF-TrFE)/PEDOT:PSS composite film with NII, (b) P(VDF-TrFE) with mental electrode without NII.

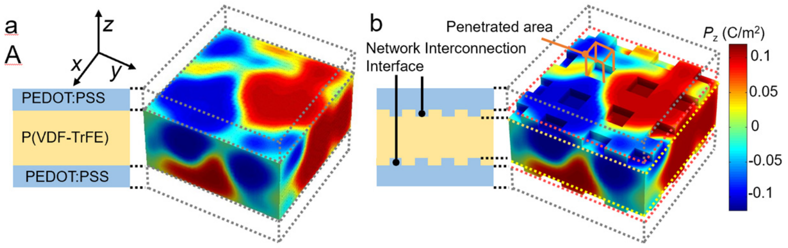

Fig. S5 Phase field simulation of the polarization distribution of P(VDF-TrFE) in the P(VDF-TrFE)/PEDOT:PSS structure (a) without and (b) with the NII structure.

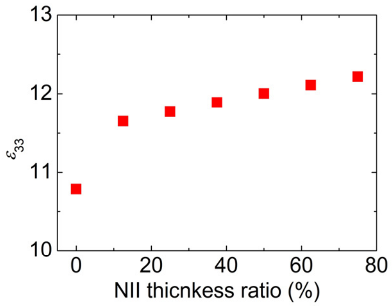

Fig. S6 Calculated dielectric coefficient of the P(VDF-TrFE)/PEDOT:PSS composite film as a function of the NII thickness ratio.

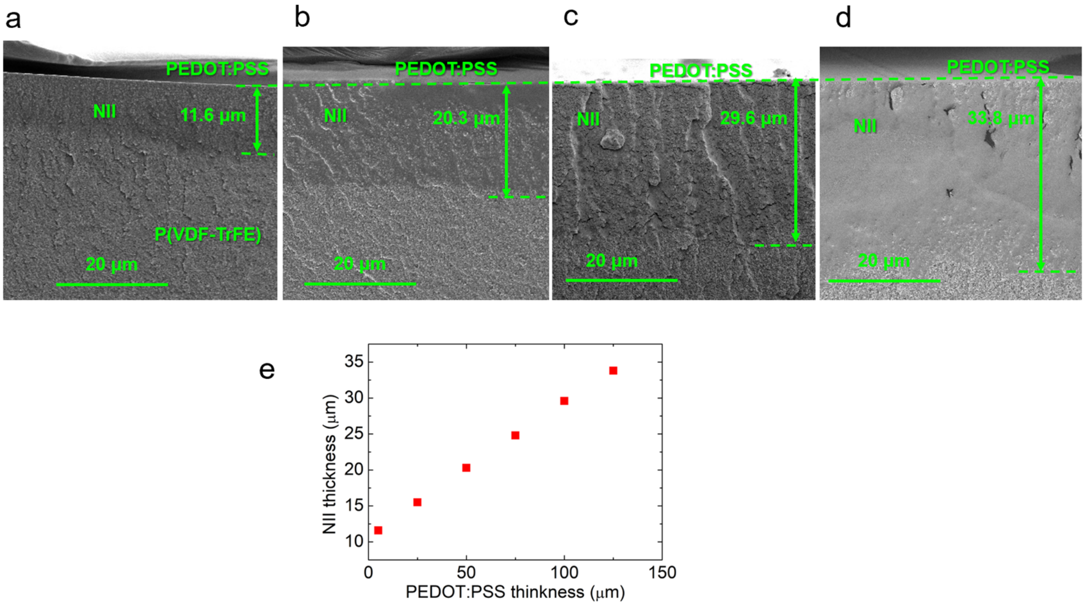

Fig. S7 Cross-sectional SEM images of PEDOT:PSS/P(VDF-TrFE) composite film with different PEDOT:PSS thicknesses: (a) 5 µm, (b) 50 µm, (c) 100 µm, and (d) 125 µm. (e) NII thickness as a function of PEDOT:PSS thickness.

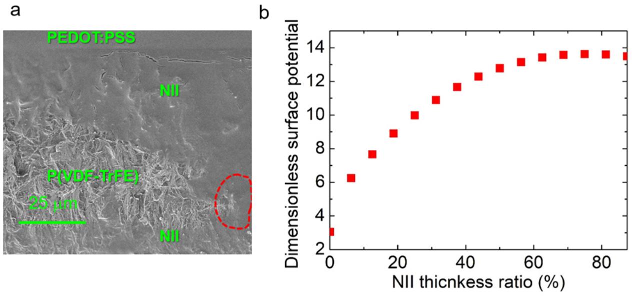

Fig. S8 The microstructure and surface potential of composite film. (a) Cross-sectional SEM images of PEDOT:PSS/P(VDF-TrFE) composite film with NII contacted. (b) Simulated dimensionless surface potential as a function of NII thickness ratio.

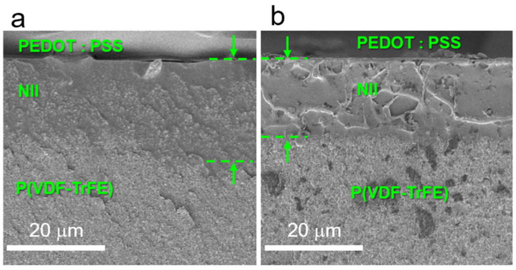

Fig. S9 Cross-sectional SEM images of PEDOT:PSS/P(VDF-TrFE) composite films prepared using (a) DMSO (5 vol%) and (b) DMF solution (5 vol%).

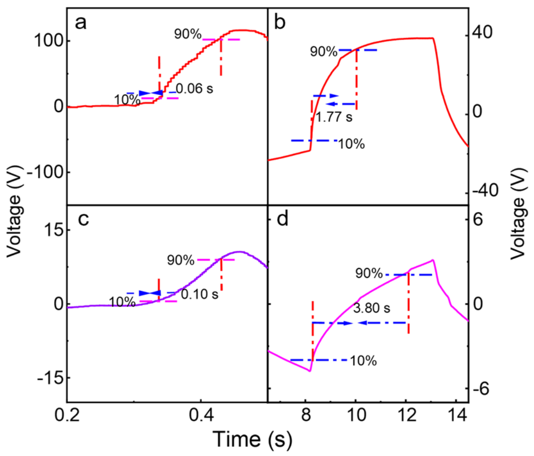

Fig. S10 Response time of output signal of P(VDF-TrFE) film induced by mechanical stimulus with (a) PEDOT:PSS, (b) Au electrode. Response time of output signal induced by thermal stimulus with (c) PEDOT:PSS, (d) Au electrode.

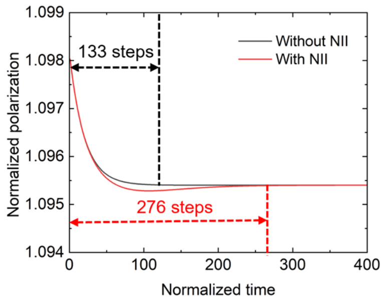

Fig. S11 Domain revolution of P(VDF-TrFE) film with and without NII as a function of normalized time obtained from phase-field simulation.

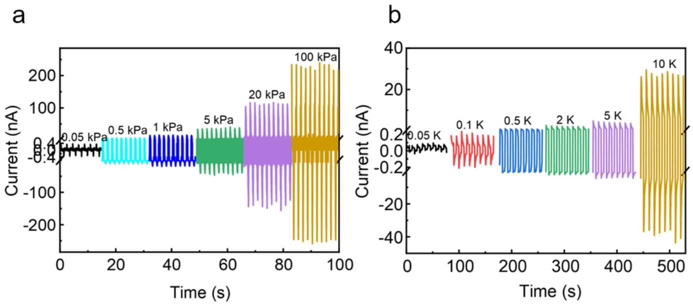

Fig. S12 Current of the composite film as a function of the (a) pressure and (b) temperature, respectively.

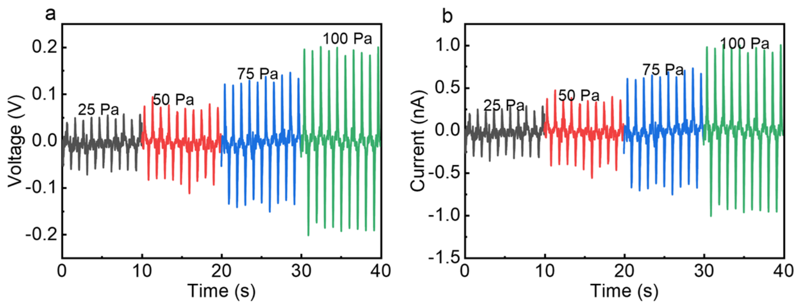

Figure S13 Piezoelectric (a) voltage and (b) current of the composite film as a function of the pressure of 25, 50, 75 and 100 Pa.

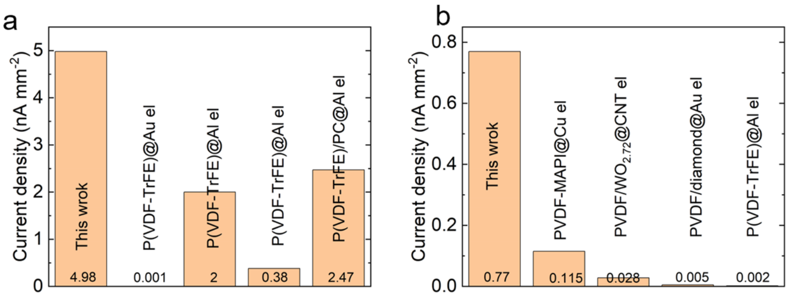

Figure S14 Comparison of (a) piezoelectric and (b) pyroelectric current density between this work and previously reported devices[5-12].

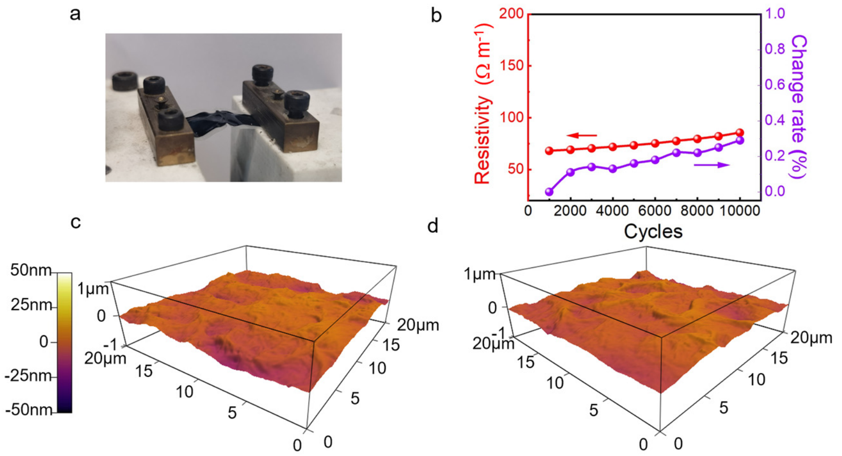

Fig. S15 PEDOT:PSS electrode durability test. (a) Home made bending test system. (b) Variation in the resistivity of the composite film as a function of the bending cycle at a radius of curvature ≤ 5.3 mm. Atomic force microscopy scans of the conductive packing layer surface: (c) surface morphology image when just prepared and (d) surface morphology image after 1000 folding cycles.

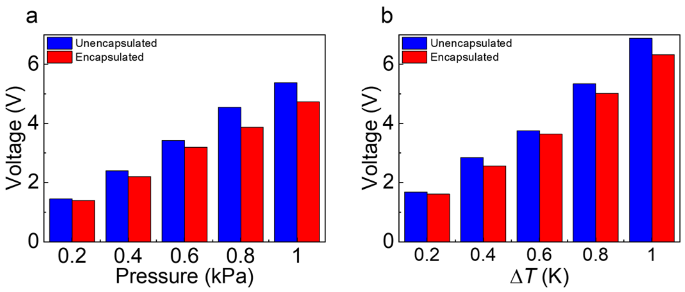

Fig. S16 Comparison of (a) pressure and (b) temperature sensor performance of the devices with and without PDMS encapsulation.

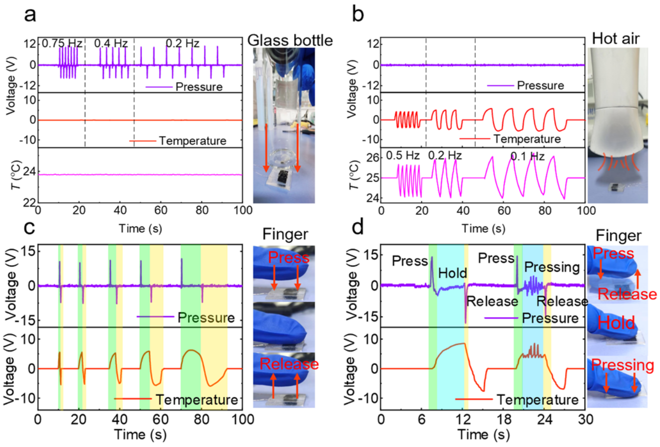

Fig. S17 (a) Single-mode response of the sensor to contact with a glass bottle. (b) Single-mode response of the sensor to hot air (contactless heating). (c) Dual-mode response of the sensor to instantaneous finger contact. (d) Dual-mode response of the sensor to different modes of static contact: (up) “press-hold-release” and (down) “press-pressing-release”. In the former, a finger is pressed on the device, held for 5 s, and then released. In the latter, a finger is pressed on the device, pressure is maintained for 5 s, and the finger is released. The hold and pressure phases of finger contact produce different pyroelectric and piezoelectric signals.

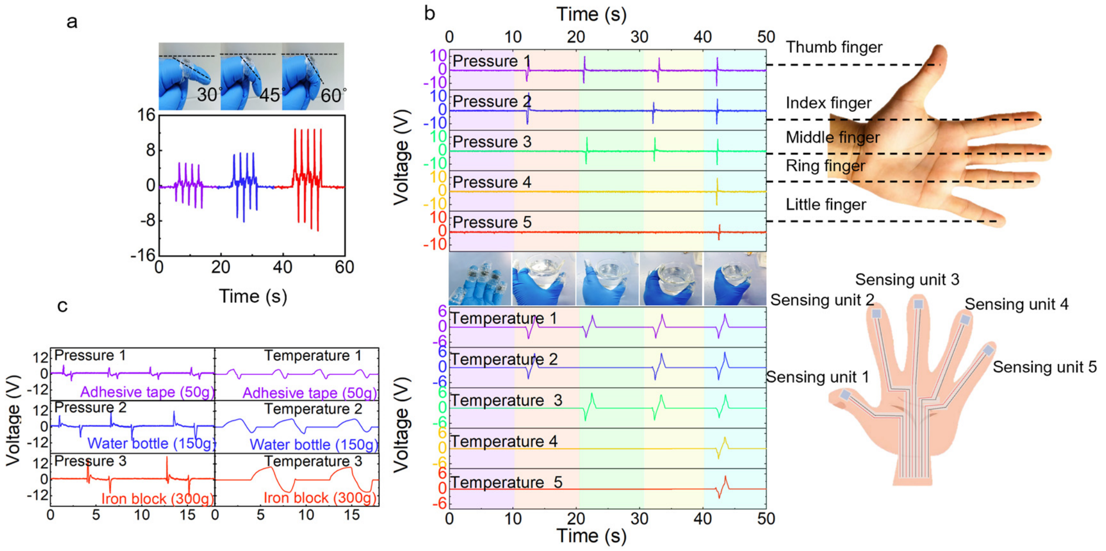

Fig. S18 (a) Strain-induced response of the sensor to finger flexion at angles of 30°, 45°, and 60°. (b) Sensor glove-based sensor array, temperature and pressure responses in contact mode. This figure shows the signals generated by the different fingers of the subject when holding a glass of water (27 °C). (c) Variation of temperature- and pressure-induced voltage as a function of the weight of the object carried.

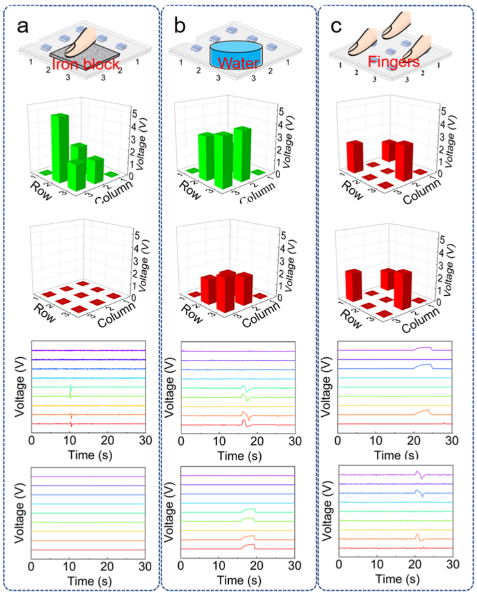

Fig. S19 Mapping images of the sensor system in contact with (a) an iron block, (b) water, and (c) fingers.

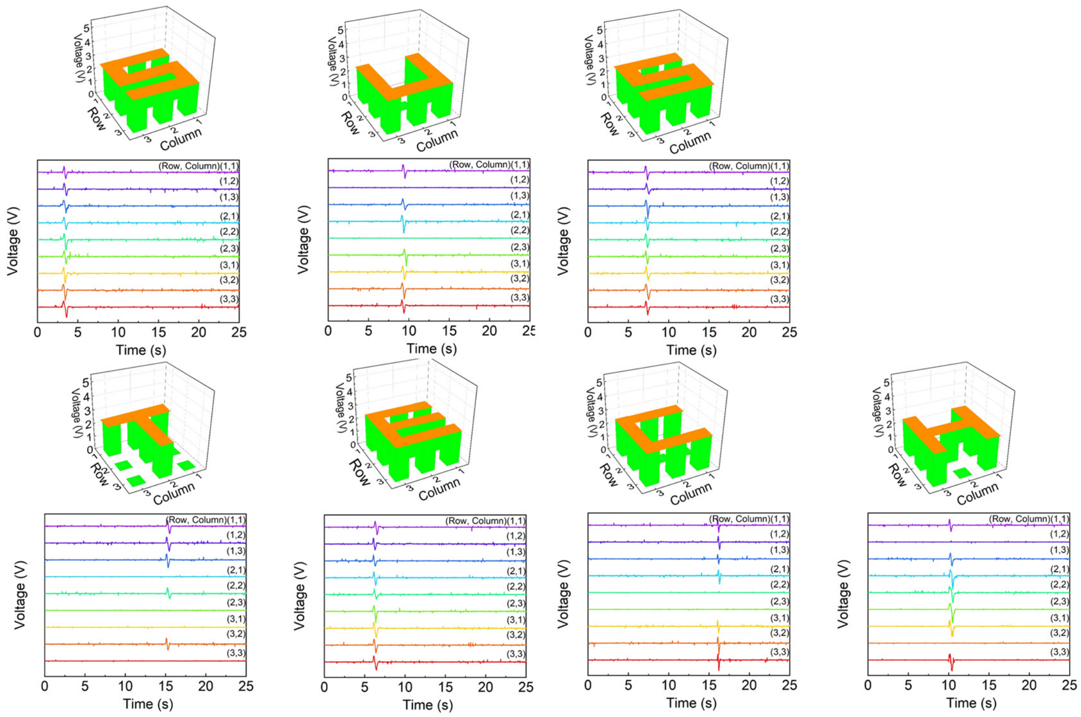

Fig. S20 Spatial mapping capabilities of multimode sensor arrays: “SUSTECH” letter mapping, and response curves of individual composite films of the letter-mold-pressed composite film array. The different cell responses observed in this image prove that the sensor array can map the dispersion of tactile sensations.

Tables

Table S1. The d_{33} of P(VDF-TrFE) films without and with different electrodes.

Structure	Sample #1	Sample #2	Sample #3	Mean	Standard Deviation
Without electrode	18	20	20	20	2
Au electrode	15	16	22	17.7	3.8
Ag electrode	17	18	21	18.7	2.1
Cu electrode	15	18	22	18.3	3.5
PEDOT:PSS electrode	86	85	83	84.7	1.5

Table S2. Measured data used to calculate the pyroelectric coefficient in the P(VDF-TrFE)/PEDOT:PSS composite film.

p (μC $m^{-2}K^{-1}$)	I (nA)	dT/dt	A (cm^2)
27.8	1.8	2.59	1.44
59.6	4.46	0.52	1.44
67.5	7.1	0.73	1.44
70.4	23.2	2.29	1.44
94.7	60	4.4	1.44

Table S3. The d_{33} the P(VDF-TrFE) films with PEDOT:PSS electrode as a function of NII thickness.

DMSO (Vol%)	Thickness (80 μm)	Thickness (130 μm)	Thickness (180 μm)	Thickness (230 μm)
5	20	30	42	62
10	20	35	44	69
15	20	39	64	74
20	20	45	71	86
25	20	25	21	15
30	20	13	9	2

Table S4. Piezoelectric and pyroelectric voltage (peak-to-peak) as a function of pressure and temperature.

Pressure (KPa)	Voltage (V)	Temperature (°C)	Voltage (V)
0.07	0.37	0.02	0.11
0.1	0.42	0.05	0.34
0.15	0.61	0.07	0.44
0.2	0.66	0.09	0.55
0.25	0.98	0.1	0.60
0.3	1.19	0.2	1.61
0.35	1.50	0.3	1.77
0.4	1.75	0.4	2.57
.45	1.88	0.5	3.42
0.5	2.06	0.6	3.64
1	3.14	0.7	4.17
1.5	4.13	0.8	5.02
2	5.40	0.9	5.98
2.5	6.61	1	6.32
3	7.99	2	12.99
3.5	9.20	3	18.59
4	10.50	4	23.32
4.5	11.24	5	32.26
5	12.01	6	37.19
10	22.12	7	43.91
15	32.20	8	47.76
20	43.40	9	58.09
25	54.20	10	64.02
30	67.50		
35	77.74		
40	89.90		
45	100.12		
50	110.40		
55	120.20		
60	132.91		
65	142.36		
70	153.81		
75	163.74		
80	175.70		
85	186.67		
90	197.10		
95	208.60		
100	220.50		

Table S5. The parameter values used in the simulation.

Parameter	Value	Parameter	Value
a_0	7.5×10^{7} J m C^{-2} K	G_{11}	9.96×10^{-10} N m^4 C^{-2}
β	-1.9×10^{12} J m^5 C^{-4}	G_{12}	0
γ	1.9×10^{14} J m^9 C^{-6}	G_{44}	4.98×10^{-10} N m^4 C^{-2}
S_{11}	3.32×10^{-10} m^2 N^{-1}	Q_{11}	0 m^4 C^{-2}
S_{12}	-1.44×10^{-10} m^2 N^{-1}	Q_{12}	3 m^4 C^{-2}
ε_0	8.85×10^{-12} F m^{-1}	T_0	307 K

Supplementary references

[1] Pan, C., Dong, L., Zhu, G., Niu, S., Yu, R., Yang, Q., Liu, Y., Wang, Z. L., High-resolution electroluminescent imaging of pressure distribution using a piezoelectric nanowire LED array. *Nat. Photonics* 9, 752-758 (2013)

[2] Spearman, C. The proof and measurement of association between two things. *Int. J. Epidemiol.* 39, 1137-1150. (2010)

[3] Goodman, L. A., Kruska,l W., Measures of association for cross classifications. *J. Am. Stat. Assoc.* 49, 732-764 (1954)

[4] Heywang, W., Lubitz, K. & Wersing, W. Piezoelectricity: Evolution and Future of a Technology. (Springer Publishing Company, Incorporated, 2008).

[5] Bhavanasi, V., Kusuma, D. Y. & Lee, P. S. Polarization orientation, piezoelectricity, and energy harvesting performance of ferroelectric PVDF-TrFE nanotubess synthesized by nanoconfinement. *Adv. Energy Mater.* 4, 1400723 (2014).

[6] Lv, F., et al. In-situ electrostatic field regulating the recrystallization behavior of P (VDF-TrFE) films with high β-phase content and enhanced piezoelectric properties towards flexible wireless biosensing device applications. *Nano Energy* 100,107507 (2022).

[7] Chai, Bin, et al. Modulus-modulated all-organic core-shell nanofiber with remarkable piezoelectricity for energy harvesting and condition monitoring. *Nano Lett.* 23, 1810-1819 (2023).

[8] Li, W. et al. High pyroelectric effect in poly(vinylidene fluoride) composites cooperated with diamond nanoparticles. *Mater. Lett.* 267, 127514,127514 (2020).

[9] Mahdi, R. I., Gan, W. C. & Abd. Majid, W. H. Hot plate annealing at a low temperature of a thin

ferroelectric P(VDF-TrFE) film with an improved crystalline structure for sensors and actuators. *Sens.* 14, 19115-19127 (2014).

[10] Kim, J. et al. High-performance piezoelectric, pyroelectric, and triboelectric nanogenerators based on P(VDF-TrFE) with controlled crystallinity and dipole alignment. *Adv. Funct. Mater.* 27, 1700702 (2017).

[11] Sultana, Ayesha, et al. Methylammonium lead iodide incorporated poly(vinylidene fluoride) nanofibers for flexible piezoelectric–pyroelectric nanogenerator. *ACS Appl. Mater. Interfaces* 11, 27279-27287 (2019).

[12] Wu, C., Chou, M., Chala, T. F., Shimamura, Y. & Murakami, R. Infrared-driven poly(vinylidene difluoride)/tungsten oxide pyroelectric generator for non-contact energy harvesting. *Compos. Sci. Technol.* 178, 26-32 (2019).
